# Analysis of the accuracy of the Wells scale in assessing the probability of lower limb deep vein thrombosis in primary care patients practice

**DOI:** 10.1186/s12959-015-0050-4

**Published:** 2015-06-04

**Authors:** Małgorzata Dybowska, Witold Z Tomkowski, Paweł Kuca, Rafał Ubysz, Adam Jóźwik, Dariusz Chmielewski

**Affiliations:** Cardio-Pulmonary Intensive Care Department, National Institute of Tuberculosis and Lung Diseases, Warsaw, Poland; Department of Radiology, Hospital for Infectious Diseases, Warsaw, Poland; Institute of Biocybernetics and Biomedical Engineering, Polish Academy of Sciences, Warsaw, Poland; Department of Orthopedic Surgery and Traumatology, HOSPITEN Hospital, Puerto del Carmen, Lanzarote, Spain

**Keywords:** Wells scale, Deep vein thrombosis, Primary care

## Abstract

**Background:**

The clinical picture of deep vein thrombosis (DVT) is nonspecific. Therefore assessment of the probability of occurrence of DVT plays a very important part in making a correct diagnosis of DVT.

The aim of our prospective study was to assess the accuracy of the Wells scale in primary care setting in diagnostic procedure of suspected deep vein thrombosis.

**Methods:**

In the period of 20 - months (from 2007 to 2009) a group of residents from one of the urban districts of Warsaw, who reported to family doctors (22 primary care physicians were involved in the study) with symptoms of DVT were assessed on the probability of occurrence of deep vein thrombosis using the Wells scale. Family doctors were aware of symptoms of DVT and inclusion patients to this study was based on clinical suspicion of DVT.

Patients were divided into three groups, reflecting probability of DVT of the lower limbs.

To confirm DVT a compression ultrasound (CUS) test was established.

We analyzed the relationship between a qualitative variable and a variable defined on an original scale (incidence of DVT versus Wells scale count) using the Mann–Whitney test. Chi-square test compared rates of DVT events in all clinical probability groups.

Patient were follow up during 3 months in primary care setting.

**Results:**

In the period of 20 months (from 2007 to 2009) a total number of 1048 patients (male: 250 , female: 798 mean age: 61.4) with symptoms suggestive of DVT of the lower extremities entered the study. Among the 100 patients classified in the group with a high probability of DVT of the lower extremities, 40 (40%) patients (proximal DVT - 13; distal DVT - 27) were diagnosed with it (95% CI [30.94% -49.80%]). In the group with a moderate probability consisting of 302 patients, DVT of the lower extremities was diagnosed in 19 (6.29%) patients (95% CI [4.06% -9.62%]), (proximal DVT – 1; distal DVT - 18). Of the 646 patients with a low probability of DVT of the lower extremities distal DVT was diagnosed in 1 (0.15%) patient (95% CI [0.03% -0.87%]).

**Conclusion:**

The Wells scale used in primary care setting demonstrated a high degree of accuracy.

## Introduction

The clinical picture of deep vein thrombosis (DVT) is nonspecific, and symptoms such as pain or swelling of limbs are often found in many other diseases. Therefore assessment of the probability of occurrence of the disease plays a very important part in making a correct diagnosis of DVT.

For many years ongoing studies have sought to devise an effective scale which would allow for the accurate assessment of the likelihood of symptomatic DVT. Currently, one of the most popular and most frequently used scales in clinical practice is the Wells scale, which is shown in Table 1.

**Table 1:** **Wells scale**

**Clinical feature**	**Score**
Active cancer (treatment ongoing or within previous 6 months or palliative)	**1**
Paralysis, paresis, or recent plaster immobilization of the lower extremities	**1**
Recently bedridden for more than 3 days or major surgery, within 4 weeks	**1**
Localized tenderness along the distribution of the deep venous system	**1**
Entire leg swollen	**1**
Calf swelling by more than 3 cm when compared with the asymptomatic leg (measured 10 cm below tibial tuberosity)	**1**
Pitting edema (greater in the symptomatic leg)	**1**
Collateral superficial veins (non-varicose)	**1**
Alternative diagnosis as likely or greater than that of deep-vein thrombosis	**−2**

There is however disagreement in the literature regarding its accuracy. The scale was originally developed for doctors working in hospital emergency departments [1].

Studies evaluating the accuracy of the Wells scale in assessing the likelihood of deep vein thrombosis of the lower limbs in patients presenting themselves to primary care physicians provide conflicting data [2,3]. Hence the accuracy of Wells scale in primary care setting is not properly estimated yet.

## Methodology

The aim of our study was to assess the accuracy of the Wells scale in primary care setting. The study protocol was approved by local ethical committee (EC at the Institute of TB and Lung Diseases in Warsaw, Poland).

In the period of 20 - months (from 2007 to 2009) a group of residents from one of the urban districts of Warsaw, who reported to family doctors (22 primary care physicians were involved in the study) with symptoms of DVT were assessed on the probability of occurrence of this disease using the Wells scale [1].

Family doctors were aware of symptoms of DVT (redness, pain, edema) and inclusion patients to this study was based on clinical suspicion of DVT. Primary care physicians estimated Wells scoring and documented it.

Ongoing antithrombotic treatment due to already established diagnosis of DVT was the only exclusion criterion.

In accordance with the points allotted in evaluation of patients with suspected DVT, patients were divided into three groups, reflecting probability of DVT of the lower limbs. A points score of 0 or less indicated a low probability of occurrence of DVT, a score of 1 or 2 points indicated a moderate probability of occurrence of DVT and three or more points indicated a high probability.

Subsequently, all patients immediately (time frame between inclusion and ultrasound test was several minutes) underwent deep vein ultrasonography of the lower limbs performed by radiologist in the primary care office and none of the patients was referred to the hospital. The ultrasound was performed using Vivid 3 S/N 6452 General Electric Medical Systems equipment.

All patients signed agreement for ultrasonography examination.

To confirm DVT a compression ultrasound (CUS) test was established. We analyzed both the proximal portion and the distal deep venous system of the lower limbs.

We performed CUS of the proximal and calf (peroneal, anterior tibial, posterior tibial) veins according to generally accepted principles in the manner previously described [4,5]. The primary criterion for diagnosing DVT was loss of venous full compressibility. The common femoral vein and femoral vein were examined with the patient in supine position. For better vision, the lower extremities were rotated externally. The veins were evaluated as distally as possible with the transducer held in both transverse and longitudinal position. To examine the popliteal vein, the patient was either in supine or prone position with knees slightly flexed. When in prone position, the legs were supported by the examiner’s fingers. Imaging of the calf was performed in the supine position with knees flexed. External rotation was employed if necessary, especially for examination of peroneal veins. The veins were identified above the ankles and followed superiorly as far as possible. A vein was considered positive for DVT when non-compressible. A written description of the exam with photographic documentation of any lesion was analyzed by an independent adjudication committee blinded to the cluster from which the patient derived.

D-dimer concentration was not assessed in patients with suspected DVT [6]. Our research team decided to perform ultrasound of deep veins in all clinical probability groups including patients in low probability group for DVT and ruling out DVT suspicion based on D-dimer level were not necessary. The ultrasonographers were not aware of the value of Wells scale points score.

The study group of patients was observed over a three month period after CUS in primary care setting.

Patients were aware of symptoms of DVT and/or PE (dyspnea, tachypnea and chest pain) and follow up was based on the presence of symptoms of DVT and/or PE in anamnesis.

No physical examination and diagnostic procedures were performed for patients without VTE symptoms.

## Statistical analysis

We analyzed the relationship between the qualitative variable and variable defined on an ordinal scale (incidence of DVT versus Wells scale count) using the Mann–Whitney test. Chi-square tests compared rates of DVT events in all clinical probability groups. The both mentioned above test were performed using the Statistica 9 (license nr: AXAP912E538724A90-D). The confidence intervals were determined using the Wilson method described for instance in [7].

The spreadsheet with a formula for their calculation can be found on the website http://vassarstats.net/prop1.html.

## Results

In the period of 20 months (from 2007 to 2009) a total number of 1048 patients (male: 250, female: 798 mean age: 61.4) with symptoms suggestive of DVT of the lower extremities entered the study. After gathering anamnesis and a physical examination the probability of DVT of the lower extremities was assessed, using the Wells scale [1]. As a group of high probability 100 patients were classified, 302 as a moderate probability group and 646 as the low one. During the 20-month follow-up, 60 episodes of DVT of the lower extremities were diagnosed.

Among the 100 patients classified in the group with a high probability of DVT of the lower extremities, 40 (40%) patients (proximal DVT - 13; distal DVT - 27) were diagnosed with it (95% CI [30.94% -49.80%]). In the group with a moderate probability consisting of 302 patients, DVT of the lower extremities was diagnosed in 19 (6.29%) patients (95% CI [4.06% -9.62%]), (proximal DVT – 1; distal DVT - 18). Of the 646 patients with a low probability of DVT of the lower extremities distal DVT was diagnosed in 1 (0.15%) patient (95% CI [0.03% -0.87%]).

The relationship between the number of points on the Wells scale, and confirmed cases of DVT are presented in Table 2.

**Table 2:** **The relationship between the number of points on the Wells scale and confirmed episodes of DVT of the lower extremities**

**Chi-square test p <0.0001**	**Number of points on the Wells scale**		
	**≤0**	**1÷2**	**≥3**
Total number of patients	646	302	100
Without Thrombosis	645	283	60
Thrombosis	1 (0.15%)	19 (6.29%)	40 (40%)
95% confidence interval	0.03%÷0.87%	4.06%÷9.62%	30.94%÷49.80%

Table 2. The relationship between the number of points on the Wells scale and confirmed episodes of DVT of the lower extremities.

Using the Chi-square test a statistically significant dependence (p <0.0001) between the number of points on the Wells scale, and confirmed episodes of DVT of the lower extremities was found. Additionally, for the two threshold values, 0 and 2, of Wells scale, the standard accuracy measures, sensitivity, specificity, positive and negative predictive values were determined.

These accuracy measures for the threshold equal to 0 and 2 are presented in the Tables 3 and 4 respectively.

**Table 3:** **The relationship between the number of points on the Wells score threshold equal to zero points and confirmed episodes of DVT**

**Threshold on Wells scale = 0**	**Thrombosis**	**Without thrombosis**
Number of points on the Wells scale: >0	59	343
Number of points on the Wells scale: ≤0	1	645
Sensitivity: 98.33%	Positive predictive value: 14.68%	
Specificity: 65.28%	Negative predictive value: 98.85%

**Table 4:** **The relationship between the number of points on the Wells score threshold equal to 2 points and confirmed episodes of DVT**

**Threshold on Wells scale = 2**	**Thrombosis**	**Without Thrombosis**
Number of points on the Wells scale: >2	40	60
Number of points on the Wells scale: ≤2	20	928
Sensitivity: 66.66%	Positive predictive value: 40.00%	
Specificity: 93.93%	Negative predictive value: 97.89%	

**Figure 1:**
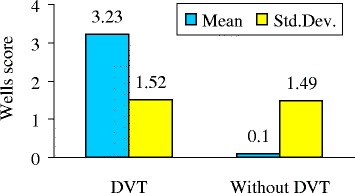
**Comparison of Wells scores in the groups with and without thrombosis.**

In the group of 60 patients diagnosed with DVT of the lower limbs the average number of points on the Wells scale was 3.23 (standard deviation 1.52), while the average number of points in patients who did not have evidence of lower limb DVT was 0.1 (standard deviation 1.49). The statistical significance of the difference between mentioned above two groups of patients was established by the Mann–Whitney test (p <0.0001). The descriptive measures of these two groups of patients are given in the Table 5 and presented in the Figure 1.

**Table 5:** **The relationship between the number of points on the Wells scale for the score threshold equal to 2 points and confirmed episodes of DVT**

**M-W test p < 0.0001**	**N**	**Median**	**Mean**	**Std. Dev.**	**Min**	**Max**
Without thrombosis	988	0	0.10	1.49	−2	7
Thrombosis	60	3	3.23	1.52	0	7

In the group of patients with negative CUS no new event of confirmed DVT occurred during the 3 month follow up period.

## Discussion

The Wells scale was originally designed as an auxiliary tool for assessing patients with symptoms suggestive of DVT in hospital Emergency/Admission Departments.

In the paper published in 2005, evaluating the usefulness of the Wells scale in assessing the probability of DVT in primary care, reference is critical as to its accuracy [2].

The results of our own work, based only on the use of the Wells scale, show a high degree of accuracy and thus confirm its usefulness in the assessment of the probability of deep venous thrombosis of the lower limbs in primary care setting.

The reason for discrepancies is the possibility of granting negative points on the Wells scale, which in a large measure determine the qualification to the individual risk groups. These points are awarded based on a subjective assessment by the examiner, and largely rely on his experience. In the original Wells and co-workers publication, doctors evaluating the scale had extensive experience in the diagnosis of VTE [1].

The previously mentioned widespread controversy in the literature, centered on the influence of subjective assessment in the classification of patients with symptoms of DVT using the Wells scale. This problem stimulated attempts to create a point scale based on objective data.

In 2006, Dutch researchers devised a point scale specifying four groups of clinical probability of patients with suspicion of DVT: very low (0–3 points), low (4–6 points), medium (7–9 points) and high (10–13 points) [6]. In this scale awarding of subjective negative points was eliminated, and an objective test based on the determination of D – dimers level was added.

The scale was evaluated in 2009 in primary health care in a group of more than 1000 patients suspected of DVT of the lower limbs [8]. Patients with 4 or more points were qualified for further ultrasound evaluation.

It is worth emphasizing that a comparable number of patients with suspicion of DVT entered our study.

Based on the results of our own work, we found a highly accurate Wells scale performance, when used by primary care physicians in assessing clinical probability of deep venous thrombosis of the lower limbs.

An important issue relates to patients with a high clinical probability of DVT. In our opinion in this particular group of patients ultrasonography of deep veins should be performed without delay. Prior determination of D-dimer level is not indicated. It should be emphasized that in the groups of patients with moderate and low clinical probability of DVT the rate of confirmation by CUS thrombosis was low and not one of the patients with negative CUS in the 3 months follow-up period expressed a new event of DVT. This very significant observation indicates the urgent need for more cost- effective procedures, with higher specificity and higher sensitivity.

To date we do not employ such a test. Nevertheless, the presence of negative D-dimer substantially reduces the necessity of CUS. However, the precision of the ruling out procedure in DVT assessment still remains inadequate due to low specificity of positive D-dimer test. Hence, waste of funds on unnecessary CUS still remains high [6,8].

There were some limitations of our study:

1. relatively low simple size of investigated group of patients,
2. no performed assessment of D-dimer concentration in patients with suspected DVT.

## Conclusions

Based on the results of our own work, the Wells scale used in primary care setting demonstrated a high degree of accuracy.

In patients with high probability of DVT assessed by Wells scoring index ultrasonography of deep veins should be performed without delay and regardless of prior determination of D-dimer level.

## Abbreviations

DVT: Deep vein thrombosis
CUS: Compression ultrasound
PE: Pulmonary embolism
VTE: Venous thromboembolism
